# Awareness of physicians and dentists in Serbia about the association between periodontitis and systemic diseases: a cross-sectional study

**DOI:** 10.1186/s12903-023-03143-3

**Published:** 2023-07-05

**Authors:** Marija Stojilković, Ivana Gušić, Dušan Prodanović, Miloš Ilić, Nevena Pecikozić, Tanja Veljović, Jelena Mirnić, Milanko Đurić

**Affiliations:** 1 Department of Dental Medicine, Faculty of Medicine, University of Novi Sad, Hajduk Veljkova 3, Novi Sad, 21000 Serbia; 2 Dentistry Clinic of Vojvodina, Hajduk Veljkova 12, Novi Sad, 21000 Novi Sad, Serbia; 3 Department of Pharmacology, Toxicology and Clinical Pharmacology, Faculty of Medicine, University of Novi Sad, Hajduk Veljkova 3, Novi Sad, 21000 Serbia; 4 Department of Pharmacy, Faculty of Medicine, University of Novi Sad, Hajduk Veljkova 3, Novi Sad, 21000 Serbia

**Keywords:** Awareness, Knowledge, Periodontitis, Non-communicable diseases, Periodontal medicine, Physicians, Dentists, Professional practice

## Abstract

**Background:**

Strong evidence supports the association between periodontitis and certain systemic diseases. The aim of the present study was to evaluate the knowledge of a group of physicians and dentists in Serbia regarding this topic and assess their professional actions to prevent and control both periodontal and systemic diseases.

**Methods:**

An anonymous self-administered structured questionnaire was sent to the available e-mail addresses of randomly selected healthcare providers working in Serbia. According to the inclusion criteria, general practitioners, specialists, general dentists, and specialists working in government hospitals and private practices in various cities in Serbia were recruited in the study. The questionnaire consisted of 17 questions divided into three parts. The first part recorded the sociodemographic characteristics of participants, the second part included questions about the clinical manifestation and etiology of periodontitis, as well as knowledge of the association between periodontitis and systemic diseases, and the third part included questions about professional procedures for the prevention and control of periodontitis and systemic diseases.

**Results:**

A total of 1301 health participants, 739 (57.8%) physicians and 562 (43.2%) dentists, were included in this cross-sectional study. Most respondents (94.7%) were aware of the association between periodontitis and general health. The highest percentage of respondents associated diabetes mellitus and periodontitis. Factors significantly associated with higher knowledge were female sex (odds ratio [OR], 1.86; 95% confidence interval [CI], 1.37–2.52; p < 0.001) and dental profession (OR, 5.86; 95% CI], 4.03–8.53; p < 0.001). Participants who had higher knowledge score were more likely to ask their patients about gum/systematic health (p < 0.001) and refer them to dentists/physicians (p < 0.001).

**Conclusions:**

It was concluded that compared to the group of dentists, the group of physicians had less knowledge of the relationship between periodontitis and systemic diseases. The female gender was significantly associated with better knowledge. A better understanding of this topic is associated with better clinical practice.

**Supplementary Information:**

The online version contains supplementary material available at 10.1186/s12903-023-03143-3.

## Background

Periodontitis (PD) is a chronic inflammatory disease of the tooth–supporting tissues [1]. The current etiology concept implies that a complex matrix of dental biofilm microorganisms is the primary cause of this disease [2]. The result of complex interactions between dental biofilm microorganisms and the host immune response is the destruction of the periodontal supportive tissues, the most common cause of tooth loss worldwide [3]. Periodontitis is the 6th most prevalent disease in the world, according to the Global Burden of Disease Study and according to the World Health Organization (WHO) Global Oral Health Report (2022), a severe form of the periodontal disease affects around 19% of the global population aged greater than 15 years [4, 5].

Patients often ignore the early stages of periodontitis, manifested as gingival bleeding upon provocation, and many of them seek help from a dentist when advanced disease signs appear. The reason for this is this disease’s relatively “silent” nature and low periodontal health awareness [6]. As periodontitis is the leading cause of tooth loss in the adult population worldwide, these patients are at risk of masticatory dysfunction, which undoubtedly affects their quality of life and self-esteem [7].

In 1891, Walter D. Miller [8] pointed out that oral health can affect systemic health and that microorganisms from the oral cavity can reach distant organs and affect them. The term “Periodontal medicine” was suggested by Offenbacher [9] in 1996, who explained the association between periodontitis and systemic health more accurately. The question arises is whether periodontitis and some systemic diseases coincidentally appear simultaneously, considering that they have many common risk factors, or whether there is a cause-and-effect relationship where these diseases can initiate/affect each other [10, 11]. Current evidence shows that periodontitis can be a risk factor for various systemic diseases, such as ischemic heart disease, type 2 diabetes mellitus, premature birth, rheumatoid arthritis, Alzheimer’s disease, and chronic kidney disease [12–18].

The association between periodontal and systemic diseases can be explained by two mechanisms – direct and indirect. The direct pathway represents a metastatic infection caused by hematogenous bacterial dissemination from periodontal tissues [19]. Since there is a loss of pocket epithelium integrity due to periodontitis, even routine daily activities such as tooth brushing, flossing, or chewing can cause bacteria and their endotoxins to transition into the bloodstream [20]. The indirect pathway represents prolonged low-grade systemic inflammation that may influence the development of comorbidities [21]. Periodontal pathogens have the potential to stimulate the production of interleukin-1 (IL-1), interleukin-6 (IL-6), tumor necrosis factor-alpha (TNF-α), and prostaglandin E2 (PGF2) and increase the levels of C-reactive protein (CRP) and fibrinogen which are known risk factors for systemic diseases [22–24].

Periodontitis can be prevented, easily diagnosed, and successfully treated and controlled through appropriate professional care and long-term secondary prevention. Therefore, as the foundation of the primary care workforce, physicians should recognize symptoms related to the gingiva, advise, and refer patients to dentists to make the correct diagnosis and start the proper therapy [7]. It is believed that improved awareness of the link between oral and systemic health among patients could increase the number of patients seeking dental care and may lead to patients’ education for optimal oral health [25–27]. On the other hand, dentists should know about the association between periodontal and systemic diseases and use that knowledge to help prevent certain systemic diseases by referring patients to competent specialists [28]. Interprofessional collaboration is needed to provide optimal patient care [29].

Serbia is a southeastern European country that, according to the 2022 population census, has almost 6.700.000 inhabitants [30]. According to the Medical and Dental Chambers lists from 2023, there are approximately 35,000 licensed physicians and 9000 licensed dentists in Serbia [31, 32]. However, these numbers refer to licensed rather than practicing physicians and dentists and may, thus, overestimate the size of the health workforce.

In Serbia, there is insufficient data on how healthcare workers are familiar with this topic. The aim of this study was to evaluate the awareness of a group of physicians and dentists in Serbia regarding the association between periodontitis and systemic diseases and assess their professional actions to prevent and control these diseases. In particular, the hypothesis of this research was that dentists have higher knowledge about periodontitis and higher awareness of the association between periodontitis and systemic diseases. The second hypothesis is that medical practitioners with more knowledge about periodontitis and its association with systemic diseases will have more professional actions to prevent and control these diseases.

## Materials and methods

### Study design and participants

This study comprised a cross-sectional study that surveyed physicians and dentists employed in various cities in Serbia. Data collection lasted six months (from April 2022 to September 2022). According to the inclusion criteria, general practitioners, specialists, general dentists, and specialists working in government hospitals and private practices in various cities in Serbia were recruited in the study. Undergraduate medical and dental students and residents were excluded from this study.

### Sampling and sample size

A minimum sample size of 652 physicians was calculated based on the margin of error (5%), confidence level (99%), response distribution (50%), and the population size of physicians (≈ 35,000). The minimum sample size for dentists (383) was calculated based on the same margin of error, confidence level and response distribution as well as for the physicians’ sample, with a difference in the population size of dentists (≈ 9000) [33].

Participants were selected using the convenience sampling method.

### Survey design and questionnaire distribution

The researchers developed an anonymous self-administered questionnaire based on evidence from the literature. The initial iteration of the questionnaire was distributed to members of the Periodontology and Oral Medicine section within the Department of Dental Medicine, Faculty of Medicine, University of Novi Sad, for further assessment. Experts specializing in relevant fields evaluated the appropriateness of the questions, and certain modifications were made based on their recommendations. These measures were implemented to ensure the instrument’s possessed adequate content, face validity, and reliability. Additionally, the research team conducted a pilot study involving a random selection of 30 physicians and 30 dentists. The feedback provided by this group was evaluated by the research team and a panel of professors, resulting in improvements to the questionnaire’s readability and clarity. Responses from those 60 participants were not included in the study.

The final version of the questionnaire was sent to e-mail addresses available on the universities’ websites, public and private practices, and through professional social networks, such as LinkedIn. The questionnaire was mailed with a cover letter informing the participants about the study’s aims and confidentiality. Participation in the study was voluntary and without financial compensation. They were allowed to withdraw from the study at any time. Google’s privacy policy research method guaranteed the anonymity of the respondents.

### Variables

The questionnaire consisted of 17 questions divided into three parts. The first part recorded participants sociodemographic characteristics, including gender (Q1), profession (Q2), years of working experience (Q4), type of practice (Q5), and main source of information about the association between periodontitis and systemic health (Q16). The second part of the questionnaire included questions about the clinical manifestation and etiology of periodontitis (Q8 and 9), and knowledge of the association between periodontitis and systemic diseases (Q10,11,12 and 13). The third part included questions about professional procedures for preventing and controlling periodontitis and systemic diseases (Q14 and 15). The questions were closed-type, with single or multiple choices. The online survey was generated such that only the answers from the fully completed survey were registered in the database, downloaded as a Microsoft Excel sheet, and included in further analysis.

When asked about the first clinical sign of periodontitis (Q8), participants answered by choosing one of the six offered answers: tooth mobility, receding gums and exposing tooth roots, bleeding gums during tooth brushing, spontaneous tooth loss, tooth discoloration, I am not sure.

The respondents were also asked about the main etiology factor for periodontitis (Q9), which was determined by five choice answers: inadequate nutrition and lack of vitamin C, heritage, poor oral hygiene and dental biofilm, smoking, I am not sure.

Claims about the association between periodontitis and general body health (Q10), the influence of systemic diseases on periodontitis, and vice versa were given (Q11 and 12). The respondents answered whether they agreed or disagreed.

For items Q8-12, a score of 1 was assigned for each correct response, and a score of 0 for each incorrect response. These five questions’ scores were summed to determine the total score for knowledge of periodontitis and its association with systemic diseases (theoretical sum:5).

Additionally, participants were asked to mark listed diseases that could be related to periodontal disease (Q13). The answers given were: ischemic heart disease, diabetes mellitus, premature birth, rheumatoid arthritis, all the diseases mentioned above, and I am not sure. The question was designed as a multiple-choice, in which participants could check one or more offered answers.

In the last part of the questionnaire, participants were asked if they ask their patients about gum health, if they are physicians, or if they ask their patients about systemic health if they are dentists (Q14). Also, physicians were asked if they refer their patients to a dentist, while dentists were asked if they refer their patients to a physician (Q15). Respondents could answer these questions with the option of yes or no.

The entire questionnaire is presented at the end of the manuscript (Supplementary file 1).

### Statistical analysis

In the statistical analysis, categorical variables were described by frequency distribution and percentages, whereas quantitative variables were described using means and standard deviations. The chi-square test was used to analyze the association between categorical variables, while the t-test was used to determine the difference in the value of a numerical variable between two independent groups.

Univariate binary logistic regression was used to determine whether independent variables (profession, sex, years of work experience, type of practice, and the main source of information) were associated with higher knowledge of periodontitis and its association with systemic disease. The regression model used the dependent variable “knowledge score” calculated in the following manner: a score of 1 was given if the participants answered correctly to Q8-Q12 from the second part of the questionnaire (correct answers are: bleeding gums while tooth brushing, poor oral hygiene and dental biofilm and answers “agree” on claims “There is an association between periodontitis and general body health”, “Some systemic diseases and conditions can lead to periodontitis” and “Periodontal disease can affect systemic health”). A score of 0 was assigned if the question was incorrectly answered. Total knowledge of periodontitis and its association with systemic diseases higher than 4 was considered „higher knowledge“ and used as a dependent variable (0-lower knowledge, 1-higher knowledge).

Thereafter, in the multivariate binary logistic regression, variables that showed a statistically significant association with the outcome variable were controlled for the possibility of other independent interferences.

Statistical analysis was done using SPSS Statistics for Windows version 24 (IBM Corporation, Armonk, NY, USA). P values that were < 0.05 was considered statistically significant.

### Ethical aspects of the research

This study was conducted in accordance with the guidelines of the Declaration of Helsinki. Ethical approval for the research was obtained from the Faculty of Medicine Ethics Committee in Novi Sad, Serbia (No.: 21/8-1-19). Every survey included an Informed Consent Statement, where participants were assured of the confidentiality of their responses using an anonymous questionnaire. They were also informed that their participation was voluntary and could stop completing the questionnaire without any consequences.

## Results

The questionnaire was sent to 1500 e-mail addresses, and 1301 participants completed it. The response rate was 86.7%. The sample included 739 physicians (56.8%) and 562 dentists (43.2%). A higher percentage of respondents were female, with less than five years of work experience, and employed in the public sector. The sociodemographic characteristics of the respondents are presented in Table 1.

**Table 1:** Sociodemographic characteristics of the respondents (n = 1301)

Characteristic	Physicians (n (%))	Dentists (n (%))	Total (n (%))
Total	739 (56.8)	562 (43.2)	1301 (100)
Gender Male Female			
	184 (24.8)	109 (19.4)	293 (22.6)
	555 (75.2)	453 (80.6)	1008 (77.4)
Years of work experience < 5 years 5–10 years 10–20 years > 20 years			
	288 (39.3)	192 (34.2)	480 (36.9)
	158 (21.5)	153 (26.9)	311 (23.9)
	157 (21.2)	149 (26.5)	306 (23.5)
	136 (18.2)	68 (12.1)	204 (15.7)
Working sector Public Private Both			
	566 (76.6)	104 (18.5)	670 (51.6)
	103 (13.9)	411 (73.3)	514 (39.5)
	70 (9.5)	47 (8.2)	117 (8.9)
The source of information University course Professional training Scientific journals Internet Other I have no knowledge			
	232 (31.4)	392 (69.8)	624 (47.9)
	71 (9.8)	105 (18.6)	176 (13.6)
	51 (7.1)	36 (6.5)	87 (6.6)
	143 (19.3)	9 (1.6)	152 (11.7)
	149 (20.1)	16 (2.8)	165 (12.7)
	93 (12.3)	4 (0.7)	97 (7.5)

The majority of respondents (68.7% physicians and 82.9% dentists) agreed that the primary clinical symptom of PD is gingival bleeding, but only slightly more than half of the assessed physicians knew that dental biofilm is the main cause of this disease (53.4% physicians vs. 90.2% dentists) (Table 2).

**Table 2:** The respondent’s knowledge of the initial clinical manifestation and etiology of periodontitis in relation to profession

Statement	Physicians (n (%))	Dentists (n (%))	Total (n (%))	p*
Bleeding gums is the first sign of periodontitis	508 (68.7)	466 (82.9)	974 (74.8)	< 0,001
Poor oral hygiene and dental biofilm are the main etiological factors of periodontitis	395 (53.4)	507 (90.2)	902 (69.3)	< 0,001

* p value calculated by using χ^2^ test for categorical variables. Significant at p < 0.05

Regardless of profession, most respondents (94.7%) were aware of an association between periodontitis and general body health. However, knowledge about this association was statistically significantly higher among dentists (χ2 = 50.913, p < 0.001, fi = 0.198) (Table 3).

**Table 3:** Respondents’ answers about the bidirectional association between periodontitis and systemic diseases in relation to profession

Statement	Physicians (n (%))	Dentists (n (%))	Total (n (%))	p*
There is an association between periodontitis and general body health	672 (90.9)	561 (99.8)	1233 (94.7)	< 0,001
Some systemic diseases and conditions can lead to periodontitis	615 (83.2)	554 (98.5)	1169 (90.2)	< 0,001
Periodontal disease can affect systemic health	563 (76.1)	510 (90.7)	1073 (82.8)	< 0,001

* p value calculated by using χ^2^ test for categorical variables. Significant at p < 0.05

**Fig. 1:**
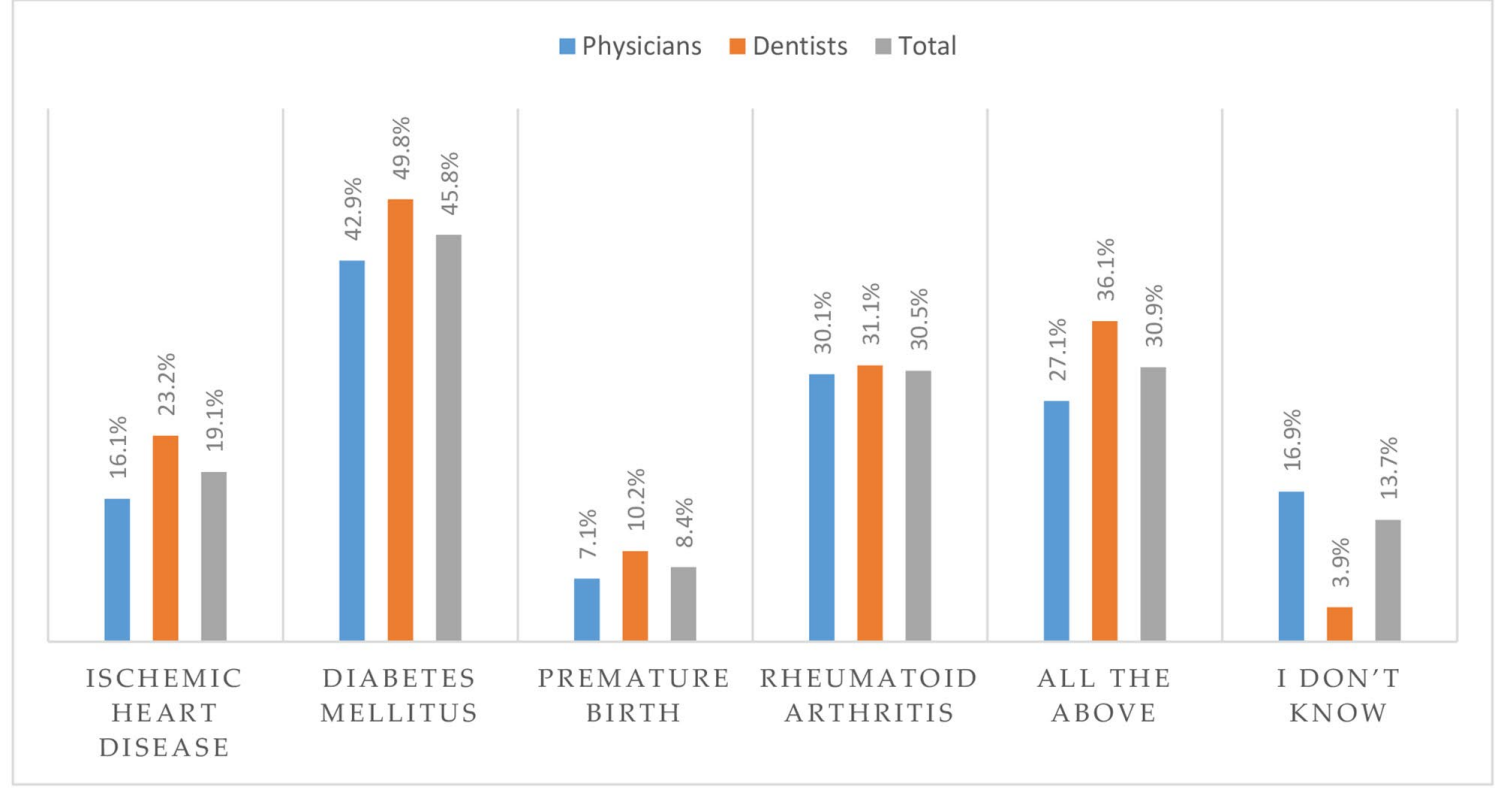
Awareness of the respondents regarding association between periodontitis and certain systemic diseases and conditions

Per the lower level of knowledge related to the previous question, physicians were significantly less informed about the connection of periodontitis with specific diseases compared to dentists. The largest number of respondents knew about the connection between periodontitis and diabetes mellitus (45.8%), whereas fewer respondents knew about the connection between periodontitis and rheumatoid arthritis (30.5%), ischemic heart disease (19.1%), or premature birth (8.4%) (Fig. 1).

The results of univariate binary logistic regression analysis showed that gender (female), profession (dentist), 5–10 years of experience, and employment in private practice were significant predictors of better knowledge of periodontitis and its association with systemic diseases, while those who had more than 20 years of experience were less likely to have higher knowledge of periodontitis and its association with systemic diseases.

A multivariate model was created to control for the mutual influence of the independent variables. Only gender and health profession remained predictors of higher knowledge of periodontitis and its association with systemic diseases (Table 4).

**Table 4:** Association between independent variables and higher knowledge of periodontitis and its association with systemic diseases

Variables	Univariate binary logistic regression				Multivariate binary logistic regression			
	**OR**	**95% CI**		**p**	**OR**	**95% CI**		p
Gender								
Male	1.00				1.00			
Female	1.978	1.504	2.601	< 0,001	1.861	1.377	2.517	< 0,001
Profession								
Physicians	1.00				1.00			
Dentists	5.288	3.922	7.130	< 0,001	5.863	4.029	8.531	< 0,001
Years of work experience								
Between 5 and 10 years of experience	1.537	1.096	2.157	0.013	1.378	0.960	1.977	0.082
Between 10 and 20 years of experience	1.031	0.749	1.418	0.853	0.921	0.649	1.306	0.643
More than 20 years of experience	0.674	0.477	0.952	0.025	0.767	0.524	1.123	0.173
Less than 5 years of experience	1.00				1.00			
Type of practice								
Private practice	2.351	1.787	3.094	< 0,001	0.767	0.548	1.129	0.193
Both	1.094	0.720	1.662	0.674	0.834	0.834	1.331	0.446
Public practice	1.00				1.00			
The source of information								
University course	1.304	0.816	2.084	0.266	1.106	0.669	1.829	0.694
Professional training	1.163	0.676	2.001	0.585	0.939	0.525	1.681	0.833
Scientific journals	1.407	0.730	2.710	0.308	1.531	0.764	3.068	0.230
Internet	1.023	0.587	1.783	0.935	0.803	0.441	1.460	0.471
Other	1.096	0.633	1.897	0.743	0.979	0.544	1.762	0.944
I have no knowledge	1.00				1.00			

Abbreviations: OR—odds ratio; CI—confidence interval

In total, 76.8% of physicians ask their patients about gum health and periodontitis, while 99.3% of dentists ask their patients about systemic health. Only 59.7% of physicians refer their patients to dentists to prevent and control periodontitis, while 97.7% of dentists advise their patients to see a competent physician. Compared with physicians, dentists ask their patients about systemic health and refer them to physicians significantly more often (p < 0.001) (Table 5).

**Table 5:** Professional actions of respondents

Question	Answers	Physicians (n (%))	Dentist (n (%))	Total (n (%))	p*
Do you ask your patients about gum health/systemic health?	Yes	568 (76.8)	558 (99.3)	1126 (86.5)	< 0.001
	No	171 (23.2)	4 (0.7)	175 (13.5)	
Do you refer your patients to physician/dentist?	Yes	441 (59.7)	549 (97.7)	990 (76.1)	< 0.001
	No	298 (40.3)	13 (2.3)	311 (23.9)	

* p value calculated by using χ^2^ test for categorical variables. Significant at p < 0.05

Both physicians and dentists, who had significantly higher knowledge score, more often ask their patients about gum/systemic health and refer them to dentists/physicians (p < 0.001) (Table 6).

**Table 6:** Professional actions in relation to the mean level of knowledge

Question	Answers	Mean score knowledge	SD	p*
Do you ask your patients about gum health/systemic health?	No	3.25	± 1.393	< 0.001
	Yes	4.23	± 0.984	
Do you refer your patients to a physicians/dentist?	No	3.25	± 1.461	< 0.001
	Yes	4.12	± 1.064	

* p value calculated by using t-test. Significant at p < 0.05

## Discussion

Numerous systemic reviews and meta-analyses have revealed an association between periodontitis and systemic diseases [12–14, 18]. In support of the evidence base, dental and medical professionals must treat the body as a whole, realizing that interdisciplinary referrals are necessary [34]. Physicians should understand the etiopathogenesis and early clinical signs of periodontitis to prevent the disease and its potential consequences. On the other hand, if dentists understand the link between periodontitis and non-communicable systemic diseases, such as ischemic heart disease and diabetes mellitus, the leading causes of death both in the world and in Serbia [35–37], they could help in their prevention. This study’s results should help assess the need for further education among health workers in this area.

Given that dentists deal with the diagnosis and treatment of periodontitis, it was expected that they would recognize the first symptom of periodontitis in a significantly higher percentage than physicians, as observed in our study. It was found that only 68% of the physicians knew that bleeding gums is the first symptom of periodontitis (Table 2). It is a striking finding that approximately one-third of those who treat systemic diseases do not know how to recognize early sign of one of the most common oral diseases. According to the curricula of medical faculties in Serbia, medical students learn about oral diseases only as part of the maxillofacial surgery course. Hence, during studies, physicians have minimal or no direct involvement in oral health. We believe this could be a possible reason for the poor understanding of the clinical manifestations of periodontitis. A similar trend was seen in the research by Narendran et al. [38], who found that approximately 73% of Indian physicians agreed that bleeding gums is the first sign of periodontitis, unlike physicians from a study conducted in Turkey [39] where a smaller percentage (59%) knew the first clinical sign of periodontitis. On the other hand, about 83% of dentists in our study showed good knowledge of the clinical manifestation of periodontitis, which is a slightly smaller percentage than dentists from a study conducted in Pakistan [40], who found that 86% of dentists were aware of the first sign of periodontitis. However, these percentages should be even higher, as all dentists need to have complete knowledge of the clinical manifestation of periodontitis.

Chronic inflammatory diseases, like periodontitis, have complex pathogenesis and multifactorial etiology. The main etiological factor is the accumulation of dental biofilms resulting from poor oral hygiene [2]. Our data showed that a bit more than half of the physicians (53%) knew the main etiological factor of periodontitis (Table 2), which is less compared to results from a study carried out in India (63%) [38]. With regard to dentists, the study by Paquette et al. [41] showed that 95% of dentists from North Carolina knew the etiology of periodontitis, which is slightly higher than the dentists in our study (90%). A lower level of knowledge regarding the etiology of periodontitis, both by physicians and dentists in our study compared to other studies, suggests that more profound education about the etiology of periodontitis is needed.

Previous studies have shown that more than 90% of physicians and dentists in countries with different health systems know that there is a connection between systemic diseases and periodontitis [39, 40, 42–44]. The results from our study are in accordance with those of previous studies and showed that more than 90% of physicians and almost all dentists (99.8%) agreed with this claim (Table 3).

The influence of various systemic diseases on the state of the periodontium has been well-researched and documented. It has been known for decades that poorly - controlled diabetes contributes to a greater intensity of inflammation and destruction of the periodontium [45, 46]. Likewise, it is well known that increased sex hormone levels during pregnancy intensify exudative gingival [47]. However, recent studies have investigated whether infections and inflammation in supporting dental tissues can influence systemic diseases and conditions [48]. Our research showed that the largest number of physicians (83%) and almost all dentists (99%) were familiar with the traditionally known and generally accepted fact that some systemic diseases may affect the periodontium. A statistically significantly smaller percentage (p < 0,001) of respondents (76.1% physicians vs. 90.7% dentists) also knew about the inverse relationship between periodontitis and systemic diseases (Table 3).

It is known that 57 different diseases and conditions are associated with periodontitis [49]. Although there is still not enough evidence that improving the health of the periodontium can influence the onset and progression of systemic diseases [50], the treatment of periodontitis should be seen as part of the prevention of systemic diseases, given that it is a risk factor that can be modified, unlike age, gender, and genetic factors. Physicians should know the risk categories of patients in which timely assessment of the periodontal condition and potential treatment of periodontitis could improve general health [51].

According to the results obtained in this study, physicians are aware of the association between periodontitis and diabetes mellitus (Fig. 1). Results from our study showed that 70% of physicians associated diabetes mellitus and periodontitis, while in other comparable studies varied from 75 to 95% [43, 52–55]. The high knowledge of the connection between these two diseases in our study and other studies is not surprising since the studies on this association are among the oldest [45]. Some studies have confirmed that periodontitis treatment contributes to better glycemic control in patients with diabetes mellitus [56, 57]. In this regard, dentists’ knowledge of the bidirectional connection between these two diseases could significantly contribute to the general health of patients with diabetes. About 80% of dentists from our research are familiar with the fact that there is a connection between periodontitis and diabetes mellitus. A similar trend was observed in a study from Saudi Arabia [53], where 80% of dentists were aware of this association. However, our results are higher than those from research conducted in North Carolina [41], where 67% of dentists agreed with this statement.

A large epidemiological study from Sweden confirmed that periodontitis could be an independent risk factor for myocardial infarction [58]. The results from our research showed that only 43% of physicians knew about this association (Fig. 1), whereas in other comparable studies, the percentage of physicians who knew about this link varied from 51 to 95% [38, 39, 43, 54, 59]. A possible explanation for the obtained results could be that ischemic heart disease does not manifest in the periodontium, so patients do not report periodontitis symptoms to physicians. Therefore, they associate ischemic heart disease with periodontitis in a small percentage. Physicians in our study showed better knowledge than physicians in a study carried out in Saudi Arabia and Kuwait (31%) [42]. However, increased awareness of the association between cardiovascular diseases and periodontitis is important to advise patients that the treatment of periodontitis can affect the level of systemic inflammatory markers and, thus, the cardiovascular disease state [60]. Better knowledge than physicians in our research was shown by dentists, 59% of whom confirmed that they knew about the connection between periodontitis and ischemic heart disease (Fig. 1). However, it is still a lower percentage compared to studies carried out in Pakistan (86%) [40] and North Carolina (72%) [41].

Interestingly, the smallest percentage of physicians (34%) in our study associated periodontitis and premature birth (Fig. 1), considering that it is one of the first conditions linked to periodontitis [61]. An even smaller percentage of those aware of this connection was found in the study conducted in Saudi Arabia and Kuwait (28%) [42]. This may be because that most physicians treat patients with diabetes mellitus and ischemic heart disease daily, while gynecologists mainly monitor pregnancy outcomes. However, this should also be improved, considering that there are studies conducted in France (75%) [59] and Pakistan (73%) [40] where physicians showed a much higher level of knowledge on this topic compared to physicians from our study. Regarding dentists, there is little information about their knowledge of the impact of periodontitis on preterm birth. In our study, 46% of dentists knew there is such a connection, slightly less compared to similar research conducted in Saudi Arabia and Kuwait [42].

The results shown in Table 4. clearly show that dentists were significantly more aware of the clinical manifestation, etiology and, association between periodontitis and systemic health compared to physicians, which is expected because they often deal with the treatment of periodontitis. In addition, medical school curricula often neglect oral health as part of general health. According to Mouradian et al. [62], it is necessary to fully implement oral health in a new curriculum for medical students, which is also an opportunity for modeling medical – dental collaboration for future practitioners. In our study, female health workers are significantly more likely to have a higher knowledge of periodontitis and its bidirectional link with systemic health. These results are similar to those of studies conducted in Kuwait [63] and New Zealand [64]. According to the results of the previous research by Ayers et al. [65], female dentists work fewer hours weekly compared to male dentists. Muhammad et al. [25] assumed that, thus, female dentists have more time to educate themselves and read the latest dental researches. Even though we did not ask our respondents about their working hours, we also, assume that the reason for the better knowledge of female participants can be related to the fewer working hours. Those with 5–10 years of work experience had the highest knowledge, among the participants. This is probably due to the combination of relatively recently acquired knowledge of the systemic–oral health connection from university course and accumulated clinical experience. Also, those with more than 20 years of experience were less likely to have higher knowledge of periodontitis and its association with systemic diseases, which means that younger generations are more familiar with this relationship than older ones. Also, medical practitioners in private healthcare facilities had more knowledge than those in the public working sector or both. The private system fosters a need to constantly innovate in one’s practice to retain the patient base and remain competitive [66], so we assume that it affects the motivation for training and additional professional education. According to our data, respondents who reported the University course and scientific journals as the main source of information had a slightly higher knowledge compared to those who chose other sources of information. Therefore, we suggest expanding oral health topics in the medical curriculum, and motivating healthcare workers to use information from published scientific articles on this topic.

Considering the high percentage of knowledge regarding the connection between periodontitis and systemic diseases (90%) among physicians, a relatively small percentage of them take specific actions in terms of referring their patients to a dentist (60%) (Table 5). The situation is better in India, where 88% of physicians ask their patients about periodontal disease and refer them to dentists [38]. The consensus report and guidelines by the European Federation of Periodontology (EFP) and International Diabetes Federation (IDF) suggest that physicians should ask people with diabetes about the prior diagnosis of periodontal disease and refer them for periodontal examination as part of their ongoing management of diabetes [67]. The EFP and World Heart Federation (WHF) also recommend that patients with cardiovascular disease should be placed on a preventive care regimen for periodontitis. If periodontitis is diagnosed, they should be managed as soon as their cardiovascular status permits [68]. Good dentist practice behavior was noted, in both respondents from our study and a study conducted in North Carolina [41], considering that there was a high percentage of respondents who ask their patients about their systemic health (99% and 90%, respectively). This is not surprising, as standard dentists’ anamnesis includes questions regarding the systemic health of the patients.

Increasing and updating medical participants’s knowledge will positively improve their attitudes and behaviors toward preventing and controlling of systemic non-communicable diseases and periodontal disease. Based on our results, physicians and dentists with significantly higher knowledge score asked their patients about periodontal/systematic health and referred them to physicians/dentists more often than health professionals with lower knowledge score (Table 6). Our results are in accordance with those obtained in a study by Fatimah Al-Habib et al. [26] who also found that physicians with increased awareness about the periodontitis-systemic link were significantly more likely to refer their patients to dentists. Increasing awareness of the association between periodontitis and systemic diseases is important so that patients can be properly advised, either by physicians or dentists.

Our findings support the importance of collaboration among health workers. To improve collaboration between physicians and dentists, there is a need to develop appropriate educational materials and professional training to enhance and update medical practitioners’ knowledge of the periodontal-systemic health association. Knowing this association, health workers could improve both the oral and systemic health of patients.

Future research could follow the implementation of health campaigns on this topic and examine their influence on the knowledge of physicians and dentists regarding the association between periodontitis and systemic diseases.

Our study had several limitations. One of them may be that medical practitioners self-reported their knowledge and professional actions, and there was no observation by the researchers. A possible limitation of such data is the social desirability bias, which is the bias toward reporting or overreporting a behavior expected by others. In addition, this study was conducted as a cross-sectional study, where only the currently observed situation could be analyzed, and tracking changes could not be observed. Finally, a limitation of this study is the use of convenience sampling to examine physicians and dentists. Since most of respondents were from larger cities, the results cannot be generalized to all medical practitioners in Serbia.

## Conclusion

The main findings of this study can be summarized as follows: knowledge regarding the clinical manifestation and etiology of periodontitis and its association with systemic disease is less in the evaluated group of physicians than in the group of dentists. Most of respondents were aware of the association between periodontitis and diabetes mellitus. Compared with dentists, physicians are less likely to ask their patients about periodontal health and refer them to the dentist. The findings of this study showed that participants who had higher knowledge of this topic were more likely to ask their patients about gum/systematic health and refer them to dentists/physicians, implying that for appropriate clinical practice, a better understanding of the link between oral and systemic diseases is necessary. This indicates the need for further education of physicians and dentists in this area to contribute to the prevention, early diagnosis, and treatment of both periodontitis and systemic diseases.

## Electronic supplementary material

Below is the link to the electronic supplementary material.

Supplementary Material 1

## Data Availability

The datasets used and analyzed during the current study are available from the corresponding author upon reasonable request.
