# Supplementary material for: Awareness of physicians and dentists in Serbia about the association between periodontitis and systemic diseases: a cross-sectional study
Source: BMC Oral Health. 2023 Jul 5;23:449. doi: 10.1186/s12903-023-03143-3 (PMC10324129; doi:10.1186/s12903-023-03143-3)
Supplement: Supplementary file 1 — Supplementary Material 1 [file 12903_2023_3143_MOESM1_ESM.docx]

Awareness of physicians and dentists in Serbia about the association between periodontitis and systemic diseases

1. Gender:

1. Female
2. Male

2. Profession:

1. General practicioner
2. Specialist in internal medicine
3. Specialist in gynecology and obstretrics
4. Specialist in another branch in medicine
5. General dentist
6. Specialist in periodontology and oral medicine
7. Specialist in another branch of dentistry

3. Which university you graduated from?

1. University of Belgrade
2. University of Novi Sad
3. University of Niš
4. University of Kragujevac
5. University of Kosovska Mitrovica

4. Years of work experience:

1. Less than 5 years
2. Between 5 and 10 years
3. Between 10 and 20 years
4. More than 20 years

5. You are employed in:

1. Government health institution
2. Private health institution
3. Both

6. In which city are you employed?

_____________________________

(In the drop-down menu we have listed all cities in Serbia)

7. How would you rate your knowledge of gum disease and periodontitis?

1. Very good
2. Good
3. Satisfactory
4. Superficially
5. I don’t know anything about periodontitis

8. In your opinion, which is the first sign of periodontitis?

1. Tooth mobility
2. Receding gums and exposing tooth roots
3. Bleeding gums while tooth brushing
4. Spontaneous tooth loss
5. Tooth discoloration
6. I am not sure

9. In your opinion, which is the main etiology factor for periodontitis?

1. Inadequate nutrition and lack of vitamin C
2. Heritage
3. Poor oral hygiene and dental biofilm
4. Smoking
5. I am not sure

10. There is an association between periodontitis and general body health.

1. Yes
2. No

11. Some systemic diseases and conditions can lead to periodontitis.

1. Yes
2. No

12. Periodontal disease can affect systemic health.

1. Yes
2. No

13. In your opinion, which systemic diseaes and conditions is periodontitis associated with? (it is possible to choose multiple answers)

1. Ischemic heart disease
2. Diabetes mellitus
3. Premature birth
4. Rheumatoid arthritis
5. All above mentioned disease
6. I am not sure

14. If you are a physicians, do you ask your patients about gum health/periodontitis? If you are a dentist, do you ask your patients about systemic diseases?

1. Yes
2. No

15. If you are a physician, do you refer your patients to a dentist in order to diagnose and possibly treat periodontitis? If you are a dentist, do you advise your patients to visit physician in order to diagnose and possibly treat systemic diseases?

1. Yes
2. No

16. What is your main source of knowledge about the association between periodontitis and systemic diseases?

1. The source of information
2. University course
3. Professional training
4. Scientific journals
5. Internet
6. Other

17. How would you rate your knowledge of the association between periodontitis and systemic diseases?

1. Very good
2. Good
3. Satisfactory
4. Superficially
5. I don’t know anything about association between periodontitis and systemic diseases
